# Eradication of metastatic melanoma through cooperative expression of RNA-based HDAC1 inhibitor and p73 by oncolytic adenovirus

**DOI:** 10.18632/oncotarget.1839

**Published:** 2014-03-21

**Authors:** Holger Schipper, Vijay Alla, Claudia Meier, Dirk M. Nettelbeck, Ottmar Herchenröder, Brigitte M. Pützer

**Affiliations:** ^1^ Institute of Experimental Gene Therapy and Cancer Research, Rostock University Medical Center, Rostock, Germany; ^2^ Helmholtz University Group Oncolytic Adenoviruses, German Cancer Research Center (DKFZ), Heidelberg, Germany

**Keywords:** Malignant melanoma, chemoresistance, oncolytic adenovirus, p73, HDAC1, apoptosis, autophagy

## Abstract

Malignant melanoma is a highly aggressive cancer that retains functional p53 and p73, and drug unresponsiveness largely depends on defects in death pathways after epigenetic gene silencing in conjunction with an imbalanced p73/DNp73 ratio. We constructed oncolytic viruses armed with an inhibitor of deacetylation and/or p73 to specifically target metastatic cancer. Arming of the viruses is aimed at lifting epigenetic blockage and re-opening apoptotic programs in a staggered manner enabling both, efficient virus replication and balanced destruction of target cells through apoptosis. Our results showed that cooperative expression of shHDAC1 and p73 efficiently enhances apoptosis induction and autophagy of infected cells which reinforces progeny production. *In vitro* analyses revealed 100% cytotoxicity after infecting cells with OV.shHDAC1.p73 at a lower virus dose compared to control viruses. Intriguingly, OV.shHDAC1.p73 acts as a potent inhibitor of highly metastatic xenograft tumors *in vivo*. Tumor expansion was significantly reduced after intratumoral injection of 3 × 10^8^ PFU of either OV.shHDAC1 or OV.p73 and, most important, complete regression could be achieved in 100% of tumors treated with OV.shHDAC1.p73. Our results point out that the combination of high replication capacity and simultaneous restoration of cell death routes significantly enhance antitumor activity.

## INTRODUCTION

Malignant melanoma is one of the most aggressive human cancers with steadily increasing incidence. Melanoma-associated mortality accounts for approximately 75% of all skin cancer related fatalities [1]. The main cause of death in melanoma patients is widespread metastasis. Metastatic disease is refractory to all current forms of therapy and has an overall poor prognosis without effective cure. Malignant progression of melanomas is associated with numerous defects in cell cycle checkpoint controls, survival and apoptotic pathways [2] that are topped by both, genetic and epigenetic events ultimately establishing the aggressive phenotype [3]. One of the central proteins of critical tumor suppressor pathways in virtually all tumor types that is able to repress melanoma initiation and progression is p53 [4, 5]. In melanoma, however, unlike most other aggressive and chemoresistant cancers, mutations in the *TP53* gene occur rarely [6]. Instead, epigenetic events including aberrant promoter methylation or histone deacetylation leading to the loss of expression of upstream activators and/or downstream effector proteins such as p14Arf or Apaf-1 that prevent the execution of apoptotic programs is frequent [7]. In this regard, previous work has shown a significant correlation between gene hypermethylation and increasing primary tumor Breslow thickness, which is associated with a high risk of metastasis development in melanoma patients [8, 9]. Given that these alterations in p53-dependent death pathways are reversible and restoration of, for example, Apaf-1 by treatment with the methylation inhibitor 5-aza-2'-deoxycytidine could rescue the observed apoptotic defects [10], reversing epigenetic changes may lead to a targeted therapy of advanced melanomas.

Another member of the p53 family that shares target gene promoters with p53 is p73. p73 exists as multiple functionally diverse protein variants that originate from alternative splicing and alternative promoter usage. Whereas the full-length isoform (TAp73) containing a transactivation domain has similar biological functions as p53, amino-terminally truncated DNp73 proteins are transactivation-deficient displaying antagonistic activities. TAp73 induces apoptosis and sensitizes cells to chemotherapy, which is blocked by DNp73 through either interfering with the binding of p53 and TAp73 to target promoters or by forming inactive heteromeric complexes with TAp73 [11]. Prognosis, disease-free survival and chemotherapeutic response in a number of human cancers are worse when the levels of the anti-apoptotic and oncogenic DNp73 variants are elevated as compared to the TA isoforms [12-14]. Characteristic for melanoma is that inhibitory DNp73 isoforms are aberrantly expressed in what are otherwise wild-type p53/p73 positive invasive and metastatic tumors [15, 16], suggesting that high levels of the oncogenic protein may contribute to the low frequency of mutations in both tumor suppressor genes in late tumor stages. We recently reported that DNp73 promotes melanoma metastasis by triggering EMT, cell migration and invasion, which is achieved by direct interference with wild-type p73-dependent stimulation of the tumor suppressor *LIMA1*/EPLIN [16]. This led to the adoption that the aggressive cancer phenotype and its lack of sensitivity to DNA damaging agents is to a large extent determined by the ratio of apoptotic to anti-apoptotic p73 proteins. DNp73 was shown to mediate drug resistance of metastatic melanoma through inhibiting p73-dependent miR-205 expression with subsequent recovery of anti-apoptotic factors like Bcl-2 [17]. Furthermore, increased sensitivity towards chemotherapy and growth inhibition of tumor xenografts in mice was achieved by specific DNp73 depletion leading to the induction of apoptotic p73 [18]. Following these observations our alternative approach is aimed to shift the balance in favor of an efficient destruction of metastatic cells by overexpressing TAp73 in conjunction with an HDAC1 inhibitor in a replicating adenovirus (Ad).

HDAC1 is responsible for the removal of acetyl moieties from histone lysine residues, leading to the exposure of the lysine's positive charge. Negatively charged DNA in turn is able to bind tightly to the histone proteins, which increases chromatin condensation. Generally, condensed chromatin is believed to impede access of transcription factors to the DNA and thus, causes a decrease in overall cellular transcription activity. Histone acetylation has also been associated with other important cellular functions like DNA repair, recombination, and chromatin assembly [19]. Moreover, deacetylation of histone H3K14 is a prerequisite for methylation of H3K9, which is typical for transcriptionally silenced regions [20]. HDAC1 not only deacetylates histones, but also other proteins, decreasing transcriptional activity of the deacetylated protein with p53 being a very prominent example [21]. Chemical inhibitors of histone deacetylases have been shown to induce apoptosis and to sensitize cells to chemotherapy or radiotherapy in cell culture and animal models [22-24]. Further, it has been shown that HDAC inhibitors are able to trigger cell death in human papilloma virus positive cells by inducing the E2F-p73 pathway [25].

Different attempts have been made to improve the efficacy of oncolytic viruses (OV) through expression of therapeutic genes in infected tumor cells [26, 27]. In order to restore epigenetically blocked apoptotic pathways in metastatic melanoma cells and, in consequence, to enhance the cytolytic effect after virus infection, we generated different OVs encoding either shRNA against histone deacetylase 1 (shHDAC1), the p73 gene, or both elements together in a timely coordinated manner.

Here, we demonstrate that the combination of ectopic p73 expression after knockdown of HDAC1 synergistically generates enhanced cytotoxicity in metastatic melanoma cells. The deactivated death programs could be restored by first lifting the epigenetic blockade to subsequently allow transactivation of p73 and p53 target genes. Most intriguingly, concurrent deacetylation activity and pro-apoptotic protein expression by the OV.shHDAC1.p73 virus enhances autophagy of infected melanoma cells that efficiently reinforces progeny production. Our results emphasize the importance of pursuing synchronous cytotoxic strategies to treat highly therapy-resistant cancers using tumor specific replicative oncolytic viruses that spares healthy cells.

## RESULTS

### Construction of oncolytic adenoviruses expressing either shRNA against HDAC1, the p73 gene, or both effectors simultaneously

**Figure 1:**
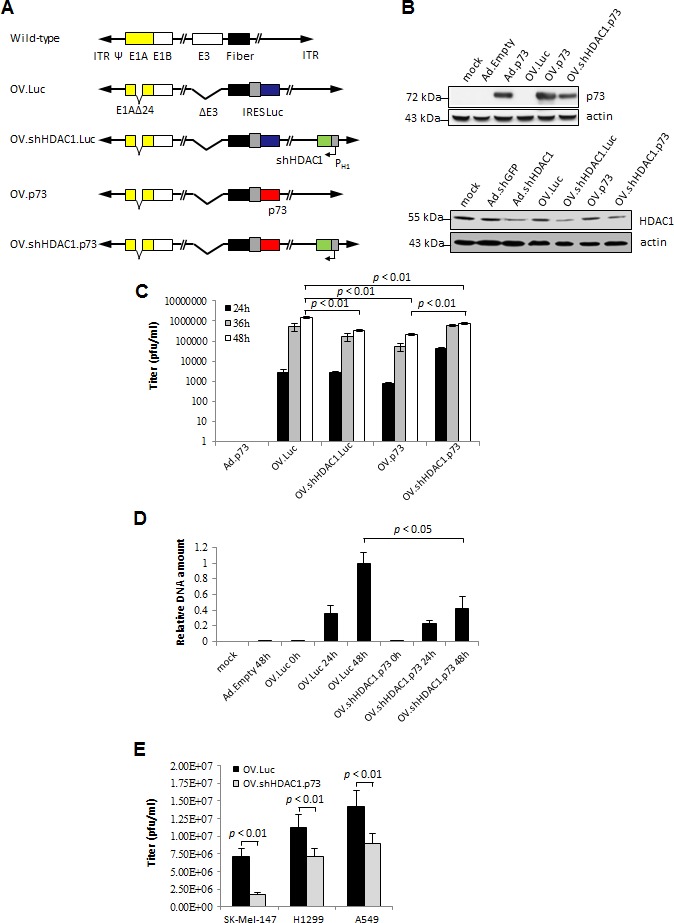
*In vitro* expression characteristics and replication efficiencies of OVs (A) Schematic representation of the OVs compared to wild-type adenovirus. A 24 bp deletion in the E1A gene (E1Adelta24) restricts OV replication to cells with inactive pRb. Furthermore, OVs harbor a deletion in the E3-region known from AdEasyI to enable efficient virus packaging after recombining the viral genomes. In OV.Luc, the luciferase gene is expressed bicistronically together with the fiber gene. OV.shHDAC1.Luc in addition, contains the gene for an shRNA directed against HDAC1 under control of the H1-promoter. In OV.p73 the luciferase gene is replaced by the p73 gene and OV.shHDAC1.p73 combines the expression of both, the p73 and the shRNA gene directed against HDAC1. (B) p73 and HDAC1 levels after infection of SK-Mel-147 cells with OVs, or replication-deficient Ad control viruses. Cells were treated with MOIs of 10. After 72 hours, protein expression was determined by Western blot. Mock: untreated cells. Actin served as loading control. (C) Burst assays were performed to measure the titers of the progeny virus at 24, 36, and 48 hours after infection of SK-Mel-147 cells with MOIs of 1. Ad.p73 served as control. (D) To compare the virus DNA content in cells after infection with OV.Luc or OV.shHDAC1.p73, SK-Mel-147 cells were infected and whole DNA was extracted 0, 24, and 48 hours post-infection. 50 ng DNA served as template for quantitative real-time PCR of the viral E2B gene. Cellular GAPDH was used for normalization. Shown are the expressional differences relative to OV.Luc 48 hours post-infection. (E) Burst assays of OV.Luc or OV.shHDAC1.p73 infected SK-Mel-147, H1299, and A549 cells 72 hours after infection at MOI 1. Ad.Empty served as control (data not shown). Data are shown as the mean ± SD of three independent experiments. *P* values of < 0.01 (C and E) and < 0.05 (D) were deemed statistically significant

In order to construct oncolytic adenoviruses with enhanced cytotoxicity, we modified the Addelta24 virus [28]. A hallmark of this virus is its replication restriction to cancerous cells due to a 24bp deletion in the *E1A* gene (E1Adelta24) which is essential for binding to the Rb protein. Due to the abolished binding of Rb to E1A, the subsequent release of E2F1 from the inhibitory E2F1-Rb complex is prevented, thus limiting the viral replication to cells with abnormal Rb control, which is commonly seen in melanoma. In clinical trials, oncolytic adenoviruses designed for cancer therapy have shown limited success [29]. Therefore, arming OVs with therapeutic genes is necessary to enhance their oncolytic efficacy. To address chemoresistance of malignant melanoma due to defects in death pathways, we aimed at apoptosis reactivation by concomitant expression of two effectors of apoptosis: p73 and an shRNA to suppress HDAC1 to reopen apoptotic routes. Figure 1A gives a schematic overview of all oncolytic viruses constructed in this study in comparison to wild-type adenovirus. The parental virus OV.Luc expresses the luciferase gene at a late stage of infection from a bicistronically transcribed RNA in conjunction with the fiber gene via an IRES sequence. OV.shHDAC1.Luc encodes in addition to the luciferase gene an shRNA directed against HDAC1 under control of the human H1-promoter. This cassette was introduced into the E4-region of the viral genome. In OV.p73, the luciferase gene in OV.Luc is replaced by the *TP73* gene. OV.shHDAC1.p73 expresses both, the shRNA against HDAC1 and p73.

### Transgene expression and replication properties of OVs *in vitro*

To validate transgene expression by the constructed OVs, highly metastatic SK-Mel-147 melanoma cells were infected with the four oncolytic viruses each at an MOI of 10. Mock infection and treatments of cells with non-oncolytic Ad-derived vectors either without a transgene (Ad.Empty), or vectors able to transduce the cells either with an shRNA against GFP (Ad.shGFP), an shRNA against HDAC1 (Ad.shHDAC1) or *TP73* (Ad.p73) were used as expression controls (Figure 1B, upper panel). As expected, strong p73 protein expression at comparable levels was observed in cells treated with the replicating and replication-deficient virus. Cells treated with OV.p73 showed a distinctly higher p73 protein level compared to OV.shHDAC1.p73, which can be explained with the recent finding that HDAC1 regulates p73 protein stability via HSP90 interaction [30]. In melanoma cells infected with viruses containing shHDAC1, the histone deacetylase level was significantly reduced via inhibiton of the HDAC1 mRNA by the specific shRNA (Figure 1B, lower panel).

Burst assays were performed on SK-Mel-147 cells to evaluate the progeny production of viruses. Cells and supernatants were collected 24, 36, and 48 hours after infection with the OVs at MOIs of 1 and the viral titers were estimated by TCID_50_ determination (Figure 1C). The low MOI was chosen to reduce re-infection events during the observation time. Within 24 hours, the OV expressing both apoptosis effectors replicated faster than the parental virus. After 48 hours, the virus titer reached almost the same level as the parental virus. Interestingly, both OVs that express either p73 or shHDAC-1 alone showed overall lower replication capacities than OV.shHDAC1.p73. From these experiments we conclude that the combination of both effectors does not significantly hamper virus replication. Additionally performed real-time PCR data revealed that replication of viral DNA at 48 hours after infection with OV.Luc was roughly 2-fold higher than in cells infected with OV.shHDAC1.p73 (Figure 1D). These data demonstrate the efficient replication characteristics of OV.shHDAC1.p73 where progeny production is comparable to the parental OV. The replication capacities of OV.Luc and OV.shHDAC1.p73 were also tested in other cancer cell lines and compared with that in SK-Mel-147. Both, the non-small cell lung carcinoma cell line H1299 and A549 adenocarcinoma cells were infected, and progeny assays were performed 72 hours post-infection. In all cell lines tested OV.Luc progeny production was higher than that of OV.shHDAC1.p73 basically reflecting the data shown in Figure 1D (Figure 1E). Virus replication is even higher in other cell lines and occurs independent of whether they express wild-type p53 or not. These data support the validity of OV.shHDAC1.p73 as a candidate for the treatment of other cancers irrespective of their p53 status.

### Enhanced cytotoxicity of OV.shHDAC1.p73 *in vitro*

**Figure 2:**
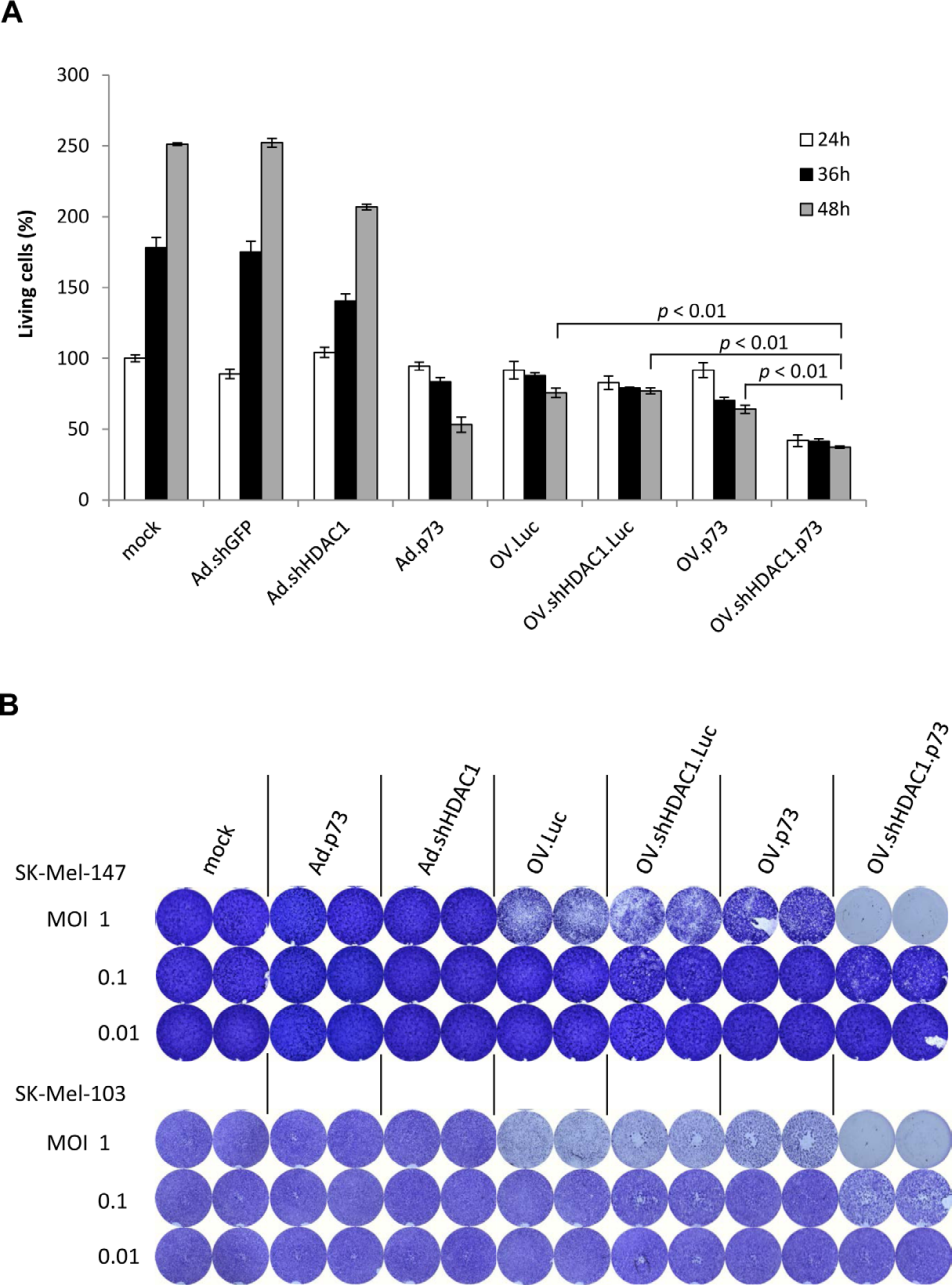
Cytotoxicity of OVs (A) Determination of living cells in percent by trypan blue exclusion at 24, 36, and 48 hours in cultures of SK-Mel-147 cells after infection with OVs at MOI 10. Cells transduced with Ad-derived vectors served as controls. The number of living cells in mock-treated cultures 24 hours after treatment was set as 100 percent. Data represent the mean ± S.D. of three independent experiments. (B) Crystal violet staining of formaldehyde-fixed SK-Mel-147 and SK-Mel-103 cells after infection of 1 × 10^4^ at MOIs of 1, 0.1, and 0.01 to demonstrate dose-dependent cytotoxicity. *P* < 0.01 is statistically significant.

In addition to their replication capabilities in tumor cells, the cytotoxic functions exhibited by OVs play a vital role to consider them as therapeutic agents. Infection with OV.shHDAC1.p73 at MOI 10 resulted in a significantly lower viability of SK-Mel-147 cells compared to all other viruses examined (Figure 2A). Strikingly, the percentage of cell viability 48 hours after infection with OV.shHDAC1.p73 decreased to 37%, in comparison to 76% for OV.Luc. In contrast, infection with OV.shHDAC1.Luc and OV.p73 reduced viability only to values comparable to OV.Luc or slightly below. These results clearly demonstrate that the OV expressing both effector genes has the highest cytotoxic potential in these metastatic melanoma cells.

These data were confirmed by dose-dependent cytotoxicity assays. As shown in Figure 2B, infection of two distinct highly aggressive melanoma cell lines with OV.shHDAC1.p73 at MOI 1 was sufficient to achieve 100% cytotoxicity within 7 days, whereas the cytotoxic potential of the other viruses was significantly lower. Moreover, cells infected with the virus expressing both effectors exhibited clearly visible cytotoxicity at the very low MOI of 0.1, while cells infected with any of the other viruses did not show any signs of cytopathic effects. Replication-deficient Ad vectors expressing shHDAC1 or p73 were not cytotoxic at MOI 0.1.

### Induction of apoptosis and autophagy after infection with oncolytic viruses

**Figure 3:**
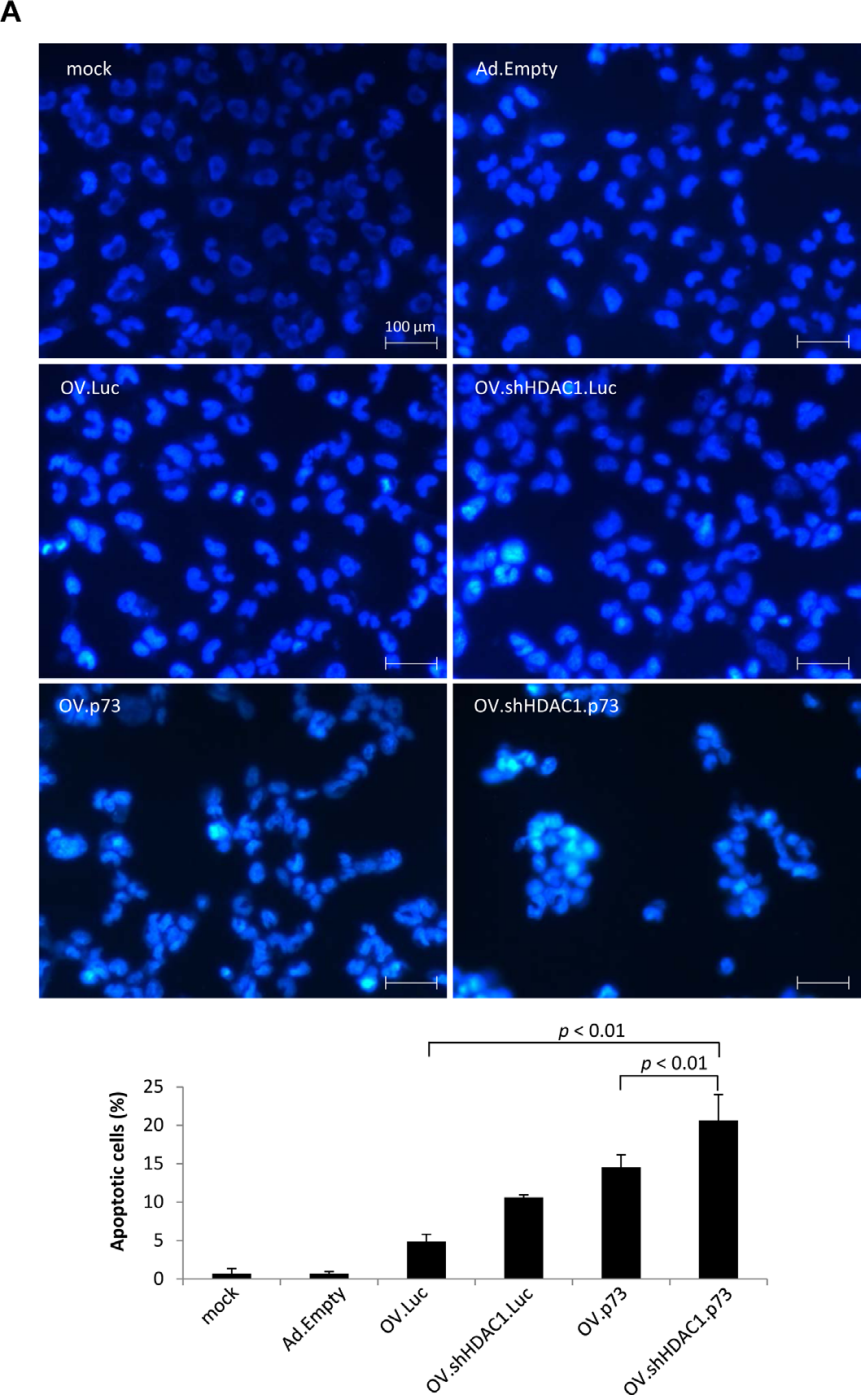

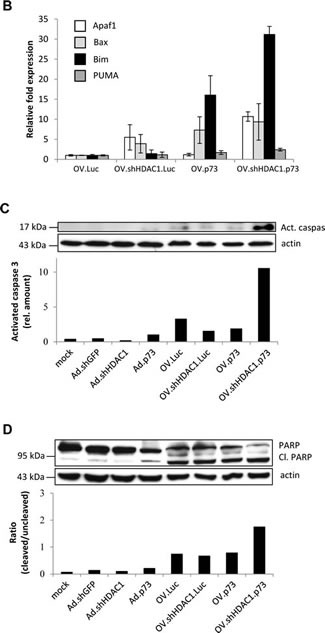
Apoptosis induction in OV-infected SK-Mel-147 cells (A) Representative photomicrographs of Hoechst 33342 stained cells exhibiting characteristic chromatin condensation after infection with OVs at MOIs of 2, 24 hours (upper panel). Size bar = 100 μm. The bar graph represents the percentages of apoptotic cells counted from each group. Data are presented as the mean of 5 different fields (lower panel). *P* values of less than 0.01 were deemed statistically significant. (B) qRT-PCR data show the expression levels of Apaf-1, Bax, Bim, and PUMA at 48 hours after infection. Data are the mean ± SD of three independent experiments. Student's t-test indicates statistically significant differences in the expression of all four genes between OV.shHDAC1.p73 and OV.Luc; of Apaf-1, Bim, and PUMA for OV.shHDAC1.p73 vs. OV.shHDAC1.Luc; and Apaf-1 plus Bim for OV.shHDAC1.p73 vs. OV.p73 (*p* < 0.05). (C) Western blot shows the increase in protein levels of active cleaved caspase 3 (Act.) 72 hours after infection of cells with OVs at MOI 10. Non-replication Ad viruses were used as controls and actin served as loading control. Based on densitometric analysis of Western blot, the bar graph shows the relative expression of activated caspase 3. (D) Increase in protein levels of active cleaved forms of PARP (Cl.) in cells infected and quantitated as in C. Bar graphs indicate the ratios of cleaved to uncleaved PARP. The experiment shown in C and D was repeated three times.

As previous reports demonstrated increased apoptosis after HDAC1 inhibition [31] or p73 overexpression [32] in melanoma cells, the capability of our oncolytic viruses to induce apoptosis was analyzed. Hoechst 33342 staining after 24 hours of cells infected at MOIs of 2 yielded the highest rate of apoptotic cells when treated with OV.shHDAC1.p73 (Figure 3A, upper panel). Significantly lower amounts of apoptotic cells were detected after infection with OVs expressing p73 or shHDAC1 alone (Figure 3A, lower panel). The percentage of apoptotic cells using TAp73, DNp73, p53 or shHDAC1 independently of each other expressed by a replication-deficient Ad vector is shown in Supplementary Figure 1.

To distinguish between necrosis by viral replication and apoptosis by repression of HDAC1 and overexpression of p73, we monitored the changes in expression of the pro-apoptotic genes Apaf-1, Bax, Bim, and PUMA 48 hours after infection of SK-Mel-147 cells with the oncolytic viruses by real-time PCR. All of these genes, independent of whether they are p53 or p73 targets, were significantly upregulated only in cells infected with OV co-expressing HDAC1 inhibitor plus p73 (Figure 3B). A significant increase was also observed after infection with OV.p73 except for the p53 target Apaf-1, which is epigenetically silenced in melanoma cells and solely enhanced by the virus able to knock down HDAC1. These data again support that OV.shHDAC1.p73 has the strongest capability to induce apoptosis in tumor cells.

One of the earlier events in programmed cell death initiation is cleavage of caspase 3 into its active form. Monitoring by Western blot the accumulation of activated caspase 3 in cells treated with Ad-derived vectors and infected with OVs gave another hint for enhanced apoptosis triggered by OV.shHDAC1.p73 (Figure 3C). No increase in uncleaved caspase 3 was observed after virus infection (Supplementary Figure 2). Poly(ADP-ribose)-polymerase 1 (PARP) is usually involved in DNA repair and a direct target for activated caspase 3. The intensity of PARP cleavage was confirmed and most prominent in cells infected with the replicating agent expressing both apoptosis effectors (Figure 3D) and proves once more the potential to induce apoptosis in cancer cells while replicating efficiently.

**Figure 4:**
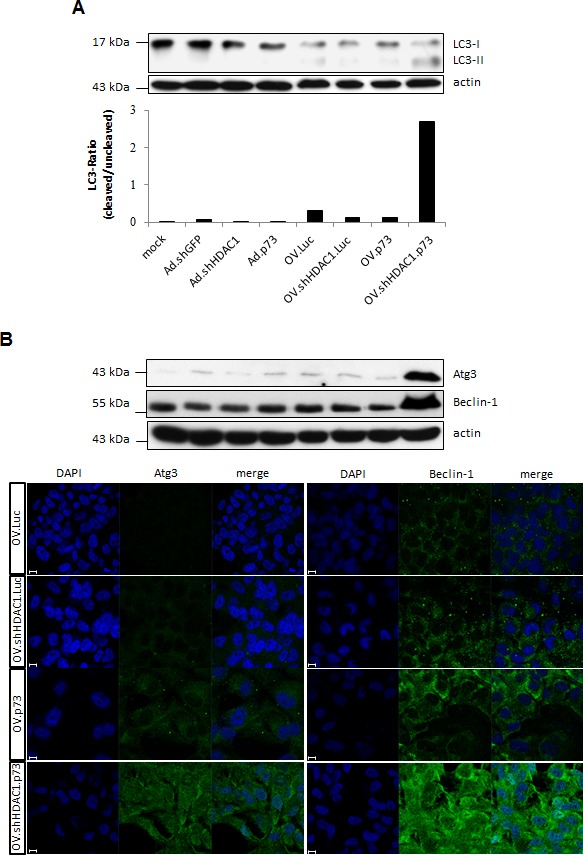
Induction of authophagy after infection with OVs (A) Western blot of LC3 as a marker of autophagy shows enhanced conversion of its LC3-I form into LC3-II (lower band) after infection with OVs. Actin was used for equal loading. Based on densitometric analysis of immunoblots the lower panel shows the ratio of LC3-II to LC3-I. This experiment was repeated three times. (B) Representative immunoblot (top) and immunofluorescence staining (bottom) of endogenous Beclin-1 and Atg3 protein expression in SK-Mel-147 cells at 48 h after infection with OVs. Beclin-1 and Atg3, green. The nuclei are evidenced by DAPI staining (blue). Size bar = 10 μm.

Macroautophagy, usually referred to as autophagy, plays an important role in cellular homeostasis and occurs as a response to stress forms such as starvation or infection by intracellularly replicating pathogens [33, 34]. It is known that infection with replication-competent adenoviruses involves induction of autophagy [35, 36] and this suggests that autophagy plays a critical role in viral structural protein synthesis likely by degrading intracellular components necessary to assemble progeny virus particles. Since autophagy might modulate the virus replication, we also investigated this catabolic mechanism by detecting the autophagy related marker LC3. Conversion of LC3-I to LC3-II is needed for autophagosome formation and the ratio of LC3-II to LC3-I represents the autophagic flux of the cell. Figure 4A shows that LC3 conversion can be observed after infection with OV.Luc and to a lesser extend after infection with OV.shHDAC1.Luc and OV.p73. Cells infected with OV.shHDAC1.p73, however, exhibit a significantly increased ratio of LC3-II to LC3-I than the three other viruses. The strongest authophagy induction through OV.shHDAC1.p73 was also confirmed by the detection of Beclin-1 and Atg3 in Western blot and immunofluorescence experiments (Figure 4B). These results indicate that not only apoptosis induction in OV.shHDAC1.p73 infected cells is distinctly higher than in cells treated with other oncolytic viruses, but also autophagy takes part in cellular disassembly. In comparison to vector controls, it becomes obvious that autophagy strongly depends on virus replication rather than overexpression of pro-apoptotic genes (Figure 4). Taken together, these observations clearly show that autophagy is induced by OV.shHDAC1.p73, which enhances viral progeny production and ultimately, cellular impairment. Further studies are needed to investigate the factors responsible for autophagy induction in OV.shHDAC1.p73 treated cells.

### Anti-tumoral effect of OV.shHDAC1.p73 *in vivo*

To examine the oncolytic effects of viruses on experimentally induced xenograft melanoma *in vivo*, we established subcutaneous tumors from SK-Mel-147 cells in nude mice. Upon injection, SK-Mel-147 induce very fast growing solid tumors and the sacrification of untreated mice becomes necessary after 2-3 weeks. Tumors were grown to a size of 100 mm^3^ and subsequently injected with three doses of 1 × 10^8^ PFU per mouse every other day.

**Figure 5:**
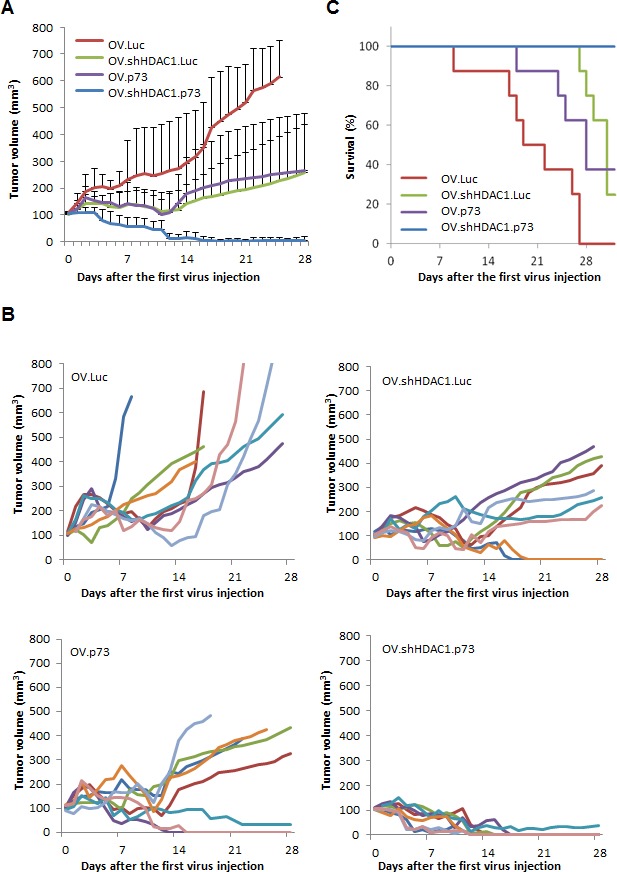
OV.shHDAC1.p73 shows improved anti-tumoral activity compared to control virus Tumors were established in NMRI nude mice by subcutaneous injection of 5 × 10^6^ SK-Mel-147 cells in the rear flanks. After reaching a volume of 100 mm^3^, tumors were treated three times (days 0, 2, and 4) with 1 × 10^8^ PFU of oncolytic virus and monitored for tumor growth. (A) Average tumor volumes in groups of mice (n=8) treated with indicated OVs. Data are mean ± SD. (B) Plots showing the changes in tumor volumes in individual mice within the groups treated with OVs as indicated. (C) Survival curves of groups of mice treated with OVs.

Figure 5 shows the tumor sizes after treatment with control OV.Luc, as well as with OV.shHDAC1.Luc, OV.p73, and OV.shHDAC1.p73. In mice receiving OV.Luc, the mean tumor volume reached 648 mm^3^ (SD 138 mm^3^) 28 days after the initial treatment. These tumor volumes were significantly higher than the mean tumor size of 264 mm^3^ (SD 214 mm^3^) and 259 mm^3^ (SD 180 mm^3^) in the groups that received OV.p73 or OV.shHDAC1.Luc, respectively. Complete tumor regression was observed in eight out of eight mice treated with OV.shHDAC1.p73 within seventeen days after treamtent was initiated. One animal retained some fibrotic tissue along the observation time. In mice treated with OV.shHDAC1 and OV.p73 the tumors progressed slower or showed some regression compared to OV.Luc. In only two mice from each of the latter groups we experienced complete tumor regression four weeks after the first treatment. One of the most common drawbacks in tumor therapy with oncolytic viruses is the recurrence of tumors, even if the immediate effect of the virus on tumor growth is significant. Survival of mice treated with oncolytic virus that coexpresses shHDAC1 and active p73 was prolonged to a statistically significant extent (C) and no signs of tumor recurrence were observed in the OV.shHDAC1.p73 group at least for 16 weeks. Altogether, despite of the rather low amount of virus used for treatment, OV.shHDAC1.p73 appears to be a potent anti-tumoral agent.

## DISCUSSION

Metastatic melanoma is a deadly cancer that fails to respond to conventional chemotherapy [37]. The basis for drug resistance in malignant melanoma is mainly dysregulation of apoptosis although other mechanisms including drug transport, detoxification, and enhanced DNA repair may also play a role [38]. Apoptotic defects have been described at multiple levels and in both major death pathways. The clinical challenge today is whether effective therapies can specifically target the pivotal triggering events and their evolution when cancer progresses [39].

p53 plays an important role in the execution of programmed cell death in tumors but, though inactivated in melanoma, gene mutations that are frequently found in other human cancers are seldom. In more than 60% of melanoma cell lines, p53 has lost its normal transcriptional activities [40] and p53-dependent pro-apoptotic genes are often underexpressed in wild-type p53-positive tumors [41, 42]. In tumor cells, downregulation of genes often results from the direct effect of histone deacetylation which blocks the transcription machinery. HDAC1, for instance, can bind to TAp73 and suppresses p73-dependent transcriptional activity [43]. Growing evidence suggests that gene expression governed by epigenetic changes is also crucial for tumor progression and that modulation of histone deacetylase activity may increase the efficiency of DNA damaging agents [44]. DNA damage-induced acetylation potentiates the apoptotic function of p73 by enhancing its ability to selectively activate transcription of pro-apoptotic genes [45]. Histone deacetylase inhibitors have been shown to exhibit an antitumoral effect in different tumor types including melanoma, and more important, have a potential to reverse epigenetic repression of tumor suppressor genes [44]. Treatment of cells with HDAC inhibitors has been reported to induce p53-independent cell cycle arrest with little or no cytotoxicity [46] and to counteract promoter repression by p73 [43]. In addition to the suppressive function of HDAC1 on apoptosis signaling in cutaneous melanoma, DNp73 isoforms that act as antagonists of wild-type p53 and p73 [11] are highly expressed in invasive and metastatic melanoma cells [15, 16]. Current data clearly implicate the imbalanced p73/DNp73 ratio co-responsible for the anti-apoptotic features of skin cancer cells [47]. Increased expression of DNp73 abrogates the apoptotic properties of p53 or p73 and switches the cells towards cancer progression [17, 48]. This opened up completely new possibilities for successful therapy of aggressive cancer phenotypes by either knocking down DNp73 or enforced p73 expression in association with removal of the epigenetic blockade. We tested here a strategy aiming at concomitant expression of an RNA-based HDAC1 inhibitor and TAp73 in the context of an oncolytic adenovirus. Gene-based virotherapy is a well known approach for anticancer treatment where tumor selectivity is achieved through deletion of viral genes critical for growth in normal cells but dispensable in tumor cells [49]. The advantage of virotherapy is that progeny viruses released after destruction of cancer cells do spread to neighbouring and distant cancer cells, and ideally eliminate the entire neoplastic tissue [50]. In the present study, we used the previously described Addelta24 harboring a 24bp deletion in the E1A gene responsible for Rb binding [28] to replicate exclusively in cancer cells.

We demonstrated that the combination of replication-competent Ad expressing shHDAC1 and p73 has a superior therapeutic effect compared to OV that solely impairs cell growth by virus replication. Our study revealed that virus encoding HDAC1 inhibitor in combination with p73 results in 100% cytotoxicity even by treating cells at a much lower concentration than the control virus. In contrast, a lesser cytotoxic effect was seen after expressing either one alone which is somehow surprising. A rational explanation for this observation is that the expression of only shHDAC1 or p73 adversely affects viral replication, as the efficacy of virus replication within cancer cells determines its cytotoxic potential. Accumulating evidence indicates that viruses actively delay apoptosis of their host cells in order to prevent the cells' disintegration prior to replication, efficient assembly, and progeny release. In this regard, previous studies reported that the chemical histone acetylase inhibitor valproic acid (VPA) acts as an antagonist of adenoviral replication [51] and might be the reason for low virus progeny production of OV.shHDAC1.Luc. Similarly, overexpression of p73 might also result in decreasing progeny due to apoptosis induction that impedes the production of mature virus particles [52]. Notably, progeny production of OV.shHDAC1.p73 was significantly higher than that of OV.p73 and OV.shHDAC1.Luc. Earlier studies showed that both, p73 and chemical inhibitors of HDAC1, like suberoylanilide hydroxamic acid (SAHA), are capable to induce autophagy in addition to apoptosis [53, 54]. The mammalian target of rapamycin (mTOR), another protein with key functions in cell growth and proliferation, for example, is able to regulate p73, and treatment with rapamycin sensitizes highly aggressive breast cancer cells to cisplatin through upregulation of TAp73 [55-57]. Our results revealed that the levels of autophagy induction measured as autophagic flux represented by the ratio of autophagosomal microtubule-associated protein 1A/1B-light chain 3 (LC3-II) to cytosolic (LC3-I) protein, differed considerably between the viruses used in this study. The conversion of LC3-I to LC3-II was lower in OV.p73 and OV.shHDAC1.Luc compared to OV.Luc infected cells. In contrast, in cells infected with OV.shHDAC1.p73, the autophagic flux increased substantially. Thus, our data support previous results indicating that adenovirus-induced autophagy correlates positively with virus replication and that autodigestion may generate nutrients to be recycled while assembling progeny particles [35]. Based on these observations, we can conclude that apoptosis and autophagy are induced more intensely in OV.shHDAC1.p73 infected than in cells treated with the other viruses. This could be a reason for the relatively higher progeny production of OV.shHDAC1.p73 compared to OV.shHDAC1.Luc and OV.p73. As previously demonstrated, an oncolytic adenovirus overexpressing beclin-1, a central protein for autophagy induction and regulation [58], had a significantly enhanced cytotoxicity compared to parental OV lacking beclin-1 in a leukemic cancer model *in vitro* and *in vivo* [59], whereas inhibition of autophagy via chloroquine resulted in enhanced oncolytic activity in glioblastoma [60], indicating that cellular prerequisites might play an important role in balancing the consequences of viral autophagy induction. Our data therefore suggest the notion that only the combination of HDAC1 knockdown and p73 overexpression enhances virus yield via a process in which autophagy is crucial. From the therapeutic perspective, a relevance of the interplay between apoptosis and autophagy for efficient elimination of melanoma cells was also reported by others [61].

To enhance apoptosis while oncolytic viruses replicate in cancer cells was the major goal of our study and indeed, the viruses we created showed substantial differences in their capacity to induce programmed cell death during replication. To differentiate between necrosis and apoptotic cellular disintegration after virus treatment, we compared cleavage of the apoptosis related proteins caspase 3 and PARP in infected cells. The hypothesis, that apoptosis rather than destruction of cells due to viral replication is the hallmark of OV.shHDAC1.p73, we compared the expression of pro-apoptotic genes influenced by p73 and HDAC1. Results demonstrated that the expression levels of Apaf-1, Bax, Bim, and PUMA were increased after infection with OV.HDAC1, OV.p73, as well as OV.shHDAC1.p73 compared to the parental OV.Luc. The expression of the above mentioned pro-apoptotic genes was significantly higher in OV.shHDAC1.p73 infected cells than in cells treated with the other viruses. Upregulation of Apaf-1 by both shHDAC1 expressing viruses also argues for the efficient restoration of endogenous p53-dependent pathways. This indicated the enhanced apoptotic capabilities of the virus expressing both, p73 and shHDAC1 while replicating efficiently.

We further confirmed the highest therapeutic efficacy of the oncolytic virus that co-expresses HDAC1 shRNA and p73 in a mouse xenograft model by treating aggressively growing melanomas with a low virus dose of 3 × 10^8^ PFU separated over three consecutive injections of 1 × 10^8^ each. Intratumoral injection of this virus caused complete tumor regression in all animals. The validity of arming oncolytic viruses with either RNA-based inhibitor of epigenetic silencing or apoptosis inducing gene was further supported in that viruses that only express shRNA against histone deacetylase 1 or the *TP73* gene lead to tumor regression in two or three out of eight mice, respectively.

Altogether, the studies reported here indicate that the combination of ectopic p73 expression with knockdown of HDAC1 generates synergistically enhanced cytotoxicity in metastatic melanoma cells. The defective death pathways could be reactivated in these tumor cells by lifting the epigenetic blockade to allow p73/p53-dependent transcription of pro-apoptotic genes using tumor specific replicative oncolytic virus. This study also showed that autophagy induced by the virus expressing both effectors plays a pivotal role for its replication. These results emphasize the importance of pursuing different cytotoxic strategies for the treatment of highly therapy-resistant cancers using oncolytic viruses. At present, patients with advanced melanoma barely profit from chemotherapeutic regimens. The reasons for nonfunctional p53/p73 pathways in melanomas and the compromised apoptotic response to chemotherapy include the inactivation of p53/p73 and their pro-apoptotic target genes by deacetylation as well as the expression of antagonistic DNp73 variants. Releasing the apoptotic genes by knocking down HDAC1 and concomitant ectopic expression of functional p73 to change the ratio of p73/DNp73 and transactivate readily available pro-apoptotic genes through highly efficient gene transfer by a replicating virus could enhance chemosensitivity. This raises the possibility that OV.shHDAC1.p73 treatment also improves the chances for successful therapy of metastatic melanoma through conventional chemotherapy.

## MATERIALS AND METHODS

### Cells and culture

HEK293, A549, H1299, SK-Mel-147, and SK-Mel-103 cell lines were cultured in DMEM with sodium pyruvate, 2 mM L-glutamine (PAA), and 10% fetal calf serum (Biochrome) with 100 μg/ml penicillin, 100 U/ml streptomycin, and 1.25 μg/ml amphotericin B (PAA) in a humidified atmosphere of 5% CO_2_ at 37°C.

### Plasmids and viruses

The plasmids modified in this study for producing oncolytic adenoviruses have been described previously [62, 63]. They comprise pVK500 which bears the adenovirus serotype 5 genome, as well as the shuttle plasmids pSdelta24 that contributes the *E1A* gene lacking the nucleotides 923–946 that encode the amino acid stretch LTCHEAGF of E1A [31], and pFiberILDE. The plasmids were used for two subsequent steps of homologous recombination carried out in BJ5183 bacteria (Stratagene) as described [64]. First, pVK500 was recombined with *Pme*I-linearized pSdelta24 or pSdelta24.shHDAC1 to produce pVK500.Sdelta24 and pVK500.Sdelta24.shHDAC1, respectively. The altered plasmids were linearized with *Swa*I and subjected to a second homologous recombination with *Pme*I-linearized pFiberILDE3 or pFiber.p73DE3 to generate pOV.Luc, pOV.shHDAC1.Luc, pOV.p73, and pOV.shHDAC1.p73, respectively. For a schematic representation of the generated oncolytic viruses see Figure 1. Plasmid pSdelta24 was used to introduce E1Adelta24 into the E1-region of the adenoviral genome. Its descendant pSdelta24.shHDAC1 was created by using the previously described newly introduced *Mlu*I/*Sal*I restriction sites between the E4 genes and the right terminal repeat (between nucleotides 35,774 and 35,775 of the Ad5 genome) of pSdelta24 [63]. This allows the additional insertion of an shRNA against HDAC1 under control of the human H1-promoter into the E4-region, without disrupting the viral replication capacity [64].The plasmid pFiberILDE3 and its descendant pFiberp73DE3 were used to either insert the luciferase or the p73 gene, respectively, into the fiber region of the virus genome. These groups of genes are then expressed bicistronically together with the fiber gene by means of an enhanced IRES sequence. Moreover, both fiber-plasmids contain the pAdEasyI E3-deletion, which was transferred together with luciferase or p73 into the adenoviral genome by homologous recombination.

The plasmid pSuper.Retro.shHDAC1 was generated by using the Oligoengine plasmid system and inserting an HDAC1 specific shRNA-sequence 5'-CGCAGATGCA GAGATTCAAC-3' [65]. The shRNA including the H1-promoter was excised from pSuper.Retro.shHDAC1 using the restriction enzymes *Eco*RI and *Bsr*GI. To generate pSdelta24.shHDAC1, the resulting fragment and the previously *Mlu*I-linearized pSdelta24 were blunted with Klenow enzyme and ligated. The luciferase gene in pFiberILDE3 was replaced with p73 by cleaving the plasmid with *Nco*I and *Xba*I. Since both restriction enzyme recognition sites existed twice in this plasmid it was necessary to remove the second *Xba*I site located outside the region of homologous recombination by partial digestion followed by blunting with Klenow enzyme and re-ligation to create pFiberILDE3X. Due to its position inside the fiber gene, the unwanted *Nco*I recognition site could not be removed in the same way. Instead, a PCR fragment was generated by using pFiberILDE3 as a template and primers to replace the *Nco*I site at the 5'-end of the luciferase gene with an *Xba*I site, pFiberNcoI-Fw 5'-CAAAACAAA AATTGGCCATGGC-3' and pFiberXbaI-Rv 5'-GCTCTAGAGTATCATCGTGTT TTTCAAAGGAAA-3'. Ligation of this *Nco*I/*Xba*I-digested PCR product with an equally digested pFiberILDE3X resulted in the plasmid pFiberDE3 retaining a unique *Xba*I restriction enzyme recognition site. The plasmid pcDNA.p73 [38] served as a PCR template to attach *Xba*I restriction sites at both ends of the p73 gene using primers p73-XbaI-Fw 5'-GCTCTAGAA TGGCCCAGTCCACCGCCAC-3' and p73-XbaI-Rv 5'-GCTCTAGATCAGGGCCCC CAGGCTCTGAC-3'. After digestion with *Xba*I, the PCR fragment was introduced into the linearized pFiberDE3 to generate pFiber.p73DE3. All plasmids were validated by PCR, restriction digestion, or sequencing.

The replication deficient adenoviral vectors Ad.Empty, Ad.shGFP, and Ad.p73 have been described previously [66]. Oncolytic viruses were produced by transfecting A549 cells with *Pac*I-digested recombined plasmids using Effectene (Qiagen) according to the manufacturer's protocol. The viruses were validated by Western blotting, subsequently enriched in A549 cells, and finally purified by two rounds of CsCl equilibrium density gradient ultracentrifugation. Physical particle concentration was determined by OD_260_ measurement (viral particles/ml) and infectious particle concentration by a standard TCID_50_ assay on HEK293 cells (plaque forming units/ml). Titers of Ad.Empty, Ad.shGFP and Ad.p73 were 2.8×10^10^ PFU/ml, 4.5×10^10^ PFU/ml and 8.9×10^9^ PFU/ml, respectively. Oncolytic viruses OV.Luc, OV.shHDAC1.Luc, OV.p73 and OV.shHDAC1.p73 had a titer of 6.3×10^9^ PFU/ml, 1.6×109 PFU/ml, 1.0×109 PFU/ml and 8.9×109 PFU/ml, respectively.

### Western blot analyses and immunofluorescence

Cells were harvested and lysed in RIPA buffer (50mM Tris-Cl, 150mM NaCl, 1% NP-40, 0.5% sodium deoxycholate, 0.1% SDS). Total protein concentration was quantified by Bradford assay (Bio-Rad) and equal amounts of protein were separated by SDS-PAGE and blotted to nitrocellulose membranes (Amersham Biosciences). Samples were probed with antibodies against p73 and PARP (both BD Bioscience), cleaved Caspase 3, HDAC1, LC3A/B (Cell Signaling), uncleaved Caspase 3 (Santa Cruz Biotechnology), Atg3 (Cell Signaling), Beclin-1 (Cell Signaling), and actin (Sigma). Secondary antibodies against mouse or rabbit IgG (Cell Signaling) were used for signal detection.

For immunofluorescence SK-Mel-147 cells were grown on coverslips and infected with OVs. At 48 h after virus infection cells were fixed in 3% paraformaldehyde, permeabilized, and stained with anti-Beclin 1 [EPR1733Y] (Abcam) or Atg3 antibody (Cell Signaling). A secondary anti-rabbit conjugated to Alexa Fluor488 (Molecular Probes) was used for visualization with a laser-scanning microscope. Cell nuclei were stained with 406-diamidino-2-phenylindole (DAPI) (5 mg/ml) (Molecular Probes).

### Burst assay

To quantify virus progeny production, 2 × 10^4^ cells were plated in 24-well plates and infected at MOI 1 in 250 μl growth medium. Two hours post-infection the medium was removed and cells were washed twice with PBS to remove unbound viruses. Then 500 μl of growth medium were added and 24, 36, 48, or 72 hours later the cells and supernatants were harvested. The viruses were released from cells through three cycles of freezing in liquid nitrogen and thawing at 37°C. Infectious virus particles were determined as plaque forming units (PFU) by the tissue culture infective method (TCID_50_) assay on HEK293 cells.

### Quantitative real-time PCR

To quantify viral DNA by qRT-PCR, 2 × 10^6^ cells were seeded onto 6-well plates and either mock treated or infected with viruses at MOI 10. After two min, 24 hours, and 48 hours cells were washed twice with PBS, trypsinized and finally resuspended in PBS. Genomic DNA was isolated using the Qiagen DNeasy Blood and Tissue Kit with an additional RNase A treatment. 50 ng of genomic DNA were then used to quantify virus DNA content in relation to cellular GAPDH. The respective primers E2B-Fw 5'-GGCATCT CGATCCAGCATATC3' and E2B-Rv 5'-CCGTGGAAAGACATGACCCT-3' were published previously [26], as well as GADPH-Fw 5'-GAGAAGTATGAC AACAGCCTCAA-3' and GAPDH-Rv 5'-TCATGGATGACCTTGGCCAG-3'. Quantification was performed using iQTM SYBR Green Supermix with the iQ5 Multicolor Real-Time PCR Detection System (Bio-Rad). Relative gene expression was calculated with the comparative Ct method using GAPDH for normalization.

To quantitiate gene expression by qRT-PCR, RNA was isolated using the RNeasy Mini Kit (Qiagen) and subsequently reverse transcribed with the Omniscript RT Kit (Qiagen). cDNA samples were mixed with iQTM SYBR Green Supermix and analyzed as described above for the quantification of viral DNA. Primers used for Apaf-1 were: Fw 5'-AACCAGGA TGGGTCACCA-3' and Rv 5'-ACTGAAA CCCAATGCACTCC-3', for Bax: Fw 5'-CACCAGCTCTG AGCAGATCAT-3' and Rv 5'-GCGGCAATC ATCCTCTGCAG-3', for Bim: Fw 5'-TCTGTT GGCAGCCTGCATTGAT-3' and Rv 5'-ATGGGAAAGCCTGCAACCAGAA-3', and for PUMA: Fw 5'-GAAGAGCAAATGAGC CAAACG-3' and Rv 5'-GGAGC AACCGGCAAACG-3', respectively.

### Living cells determination by trypan blue exclusion or crystal violet staining

The cytopathic effect within the first 48 hours after infection was determined by trypan blue exclusion cell counting. Cells (2 × 10^6^) were plated in 6-well plates and either mock treated or infected with virus at MOI 10 in 1.5 ml culture medium. After 24, 36, and 48 hours cells were trypsinized and resuspended in 500 μl PBS. Cells were then mixed with 500 μl trypan blue solution (Sigma) and subjected to cell count to determine the percentages of living cells. The cytopathic effect of the viruses at low MOI was measured by crystal violet staining. Cells (1 × 10^4^) were seeded in 48-well plates and 24 hours later either mock treated or infected with MOI 1, 0.1, or 0.01 in 0.2 ml DMEM containing 2% fetal calf serum. After 2 hours, DMEM was replaced and the cells were maintained in fresh DMEM with 10% fetal calf serum for seven days. Cells were then fixed with 3.7% formaldehyde and stained for 45 min with 1% crystal violet in 70% ethanol followed by washing with H_2_O to remove excess color. After drying, the plates were photographed.

### Hoechst 33342 staining and FACS analysis

*In situ* cytopathic effects after virus infections were visualized by Hoechst 33342 (Sigma) staining. Cells (2 × 10^6^) were seeded in 6-well plates and either mock treated or infected at MOI 2 with virus in 1.5 ml culture medium. After 24 hours, cells were stained by adding Hoechst 33342 at 1 μg/ml to the culture medium. After 20 min of incubation at 37°C cells were analyzed by fluorescence microscopy and photographed. To quantitate apoptosis induced by each OV, cells in five sections of high magnification micrographs were screened for cells with light blue nuclear staining indicating double-strand breaks and expressed as percent of total cells.

For apoptosis quantification, cells were harvested 48 hours after infection (MOI 5), fixed in 70% ethanol and stained for DNA content with propidium iodide. Analysis was performed in a FACSCalibur flow cytometer (BD Biosciences) using CellQuest software.

### *In vivo* experiments

Athymic male NMRI nu/nu mice at the age of 6 to 8 weeks were subcutaneously injected into the right flank with 5 × 10^6^ SK-Mel-147 tumor cells. Tumor volumes were monitored using calipers according to the formula V = 4π/3 × (b/2)^2^ × (a/2) (with a = length and b = width). Once the tumors had reached a mass of 100 mm^3^ usually ten to 14 days after inoculation, the mice were treated with oncolytic viruses by direct injections into the tumor. A total of three injections (each 1 × 10^8^ PFU) were administered every other day. Tumor volume developments were monitored daily and their volumes determined according to the above formula. Animals were sacrificed when tumor sizes exceeded 800 mm^3^. All experiments were performed in accordance with guidelines set by the University Institutional Animal Care and Use Committee and were approved by the local government.

### Statistical analysis

Statistical significance was calculated by paired Student's t-test. All statistical tests employed in this study were two-sided.

## SUPPLEMENTARY FIGURES



## References

[1] Garbe C, Peris K, Hauschild A, Saiag P, Middleton M, Spatz A, Grob JJ, Malvehy J, Newton-Bishop J, Stratigos A, Pehamberger H, Eggermont AM (2012). Diagnosis and treatment of melanoma. European consensus-based interdisciplinary guideline – Update 2012. Eur J Cancer.

[2] Pützer BM, Steder M, Alla V (2010). Predicting and preventing melanoma invasiveness: advances in clarifying E2F1 function. Expert Rev Anticancer Ther.

[3] Dahl C, Guldberg P (2007). The genome and epigenome of malignant melanoma. APMIS.

[4] Vazquez A, Bond EE, Levine AJ, Bond GL (2008). The genetics of the p53 pathway, apoptosis and cancer therapy. Nat Rev Drug Discov.

[5] Terzian T, Torchia EC, Dai D, Robinson SE, Murao K, Stiegmann RA, Gonzalez V, Boyle GM, Powell MB, Pollock PM, Lozano G, Robinson WA, Roop DR (2010). p53 prevents progression of nevi to melanoma predominantly through cell cycle regulation. Pigment Cell Melanoma Res.

[6] Gray-Schopfer V, Wellbrock C, Marais R (2007). Melanoma biology and new targeted therapy. Nature.

[7] Hoon DS, Spugnardi M, Kuo C, Huang SK, Morton DL, Taback B (2004). Profiling epigenetic inactivation of tumor suppressor genes in tumors and plasma from cutaneous melanoma patients. Oncogene.

[8] Bostick M, Kim JK, Estève PO, Clark A, Pradhan S, Jacobsen SE (2007). UHRF1 plays a role in maintaining DNA methylation in mammalian cells. Science.

[9] Schinke C, Mo Y, Yu Y, Amiri K, Sosman J, Greally J, Verma A (2010). Aberrant DNA methylation in malignant melanoma. Melanoma Res.

[10] Soengas MS, Capodieci P, Polsky D, Mora J, Esteller M, Opitz-Araya X, McCombie R, Herman JG, Gerald WL, Lazebnik YA, Cordón-Cardó C, Lowe SW (2001). Inactivation of the apoptosis effector Apaf-1 in malignant melanoma. Nature.

[11] Stiewe T, Theseling CC, Pützer BM (2002). Transactivation-deficient Delta TA-p73 inhibits p53 by direct competition for DNA binding: implications for tumorigenesis. J Biol Chem.

[12] Tomasini R, Mak TW, Melino G (2008). The impact of p53 and p73 on aneuploidy and cancer. Trends Cell Biol.

[13] Buhlmann S, Pützer BM (2008). DNp73 a matter of cancer: mechanisms and clinical implications. Biochim Biophys Acta.

[14] Dötsch V, Bernassola F, Coutandin D, Candi E, Melino G (2010). p63 and p73, the ancestors of p53. Cold Spring Harb Perspect Biol.

[15] Tuve S, Wagner SN, Schittek B, Pützer BM (2004). Alterations of DeltaTA-p 73 splice transcripts during melanoma development and progression. Int J Cancer.

[16] Steder M, Alla V, Meier C, Spitschak A, Pahnke J, Fürst K, Kowtharapu BS, Engelmann D, Petigk J, Egberts F, Schäd-Trcka SG, Gross G, Nettelbeck DM (2013). DNp73 exerts function in metastasis initiation by disconnecting the inhibitory role of EPLIN on IGF1R-AKT/STAT3 signaling. Cancer Cell.

[17] Alla V, Kowtharapu BS, Engelmann D, Emmrich S, Schmitz U, Steder M, Pützer BM (2012). E2F1 confers anticancer drug resistance by targeting ABC transporter family members and Bcl-2 via the p73/DNp73-miR-205 circuitry. Cell Cycle.

[18] Emmrich S, Wang W, John K, Li W, Pützer BM (2009). Antisense gapmers selectively suppress individual oncogenic p73 splice isoforms and inhibit tumor growth in vivo. Mol Cancer.

[19] Musselman CA, Lalonde ME, Côté J, Kutateladze TG (2012). Perceiving the epigenetic landscape through histone readers. Nat Struct Mol Biol.

[20] Boyle GM, Martyn AC, Parsons PG (2005). Histone deacetylase inhibitors and malignant melanoma. Pigment Cell Res.

[21] Bandyopadhyay D, Mishra A, Medrano EE (2004). Overexpression of histone deacetylase 1 confers resistance to sodium butyrate-mediated apoptosis in melanoma cells through a p53-mediated pathway. Cancer Res.

[22] Valentini A, Gravina P, Federici G, Bernardini S (2007). Valproic acid induces apoptosis, p16INK4A upregulation and sensitization to chemotherapy in human melanoma cells. Cancer Biol Ther.

[23] Gowda R, Madhunapantula SV, Desai D, Amin S, Robertson GP (2012). Selenium-containing histone deacetylase inhibitors for melanoma management. Cancer Biol Ther.

[24] Groselj B, Sharma NL, Hamdy FC, Kerr M, Kiltie AE (2013). Histone deacetylase inhibitors as radiosensitisers: effects on DNA damage signalling and repair. Br J Cancer.

[25] Finzer P, Krueger A, Stöhr M, Brenner D, Soto U, Kuntzen C, Krammer PH, Rösl F (2004). HDAC inhibitors trigger apoptosis in HPV-positive cells by inducing the E2F-p73 pathway. Oncogene.

[26] Wong HH, Lemoine NR, Wang Y (2010). Oncolytic Viruses for Cancer Therapy: Overcoming the Obstacles. Viruses.

[27] Kaufmann JK, Nettelbeck DM (2012). Virus chimeras for gene therapy, vaccination, and oncolysis: adenoviruses and beyond. Trends Mol Med.

[28] Fueyo J, Gomez-Manzano C, Alemany R, Lee PS, McDonnell TJ, Mitlianga P, Shi YX, Levin VA, Yung WK, Kyritsis AP (2000). A mutant oncolytic adenovirus targeting the Rb pathway produces anti-glioma effect in vivo. Oncogene.

[29] Vasey PA, Shulman LN, Campos S, Davis J, Gore M, Johnston S, Kirn DH, O'Neill V, Siddiqui N, Seiden MV, Kaye SB (2002). Phase I trial of intraperitoneal injection of the E1B-55-kd-gene-deleted adenovirus ONYX-015 (dl1520) given on days 1 through 5 every 3 weeks in patients with recurrent/refractory epithelial ovarian cancer. J Clin Oncol.

[30] Zhang J, Xu E, Chen X (2013). TAp73 protein stability is controlled by histone deacetylase 1 via regulation of Hsp90 chaperone function. J Biol Chem.

[31] Suzuki K, Alemany R, Yamamoto M, Curiel DT (2002). The presence of the adenovirus E3 region improves the oncolytic potency of conditionally replicative adenoviruses. Clin Cancer Res.

[32] Tuve S, Racek T, Niemetz A, Schultz J, Soengas MS, Pützer BM (2006). Adenovirus-mediated TA-p73beta gene transfer increases chemosensitivity of human malignant melanomas. Apoptosis.

[33] Schreiner S, Wimmer P, Dobner T (2012). Adenovirus degradation of cellular proteins. Future Microbiol.

[34] Boya P, Reggiori F, Codogno P (2013). Emerging regulation and functions of autophagy. Nat Cell Biol.

[35] Rodriguez-Rocha H, Gomez-Gutierrez JG, Garcia-Garcia A, Rao XM, Chen L, McMasters KM, Zhou HS (2011). Adenoviruses induce autophagy to promote virus replication and oncolysis. Virology.

[36] Jiang H, White EJ, Ríos-Vicil CI, Xu J, Gomez-Manzano C, Fueyo J (2011). Human adenovirus type 5 induces cell lysis through autophagy and autophagy-triggered caspase activity. J Virol.

[37] Soengas MS, Lowe SW (2003). Apoptosis and melanoma chemoresistance. Oncogene.

[38] Grossmann D, Altieri DC (2001). Drug resistance in melanoma: Mechanisms, apoptosis, and new potential therapeutic targets. Cancer Metastasis Rev.

[39] Nikolaou VA, Stratigos AJ, Flaherty KT, Tsao H (2012). Melanoma: new insights and new therapies. J Invest Dermatol.

[40] Houben R, Hesbacher S, Schmid CP, Kauczok CS, Flohr U, Haferkamp S, Müller CS, Schrama D, Wischhusen J, Becker JC (2011). High-level expression of wild-type p53 in melanoma cells is frequently associated with inactivity in p53 reporter gene assays. PLoS One.

[41] Avery-Kiejda KA, Bowden NA, Croft AJ, Scurr LL, Kairupan CF, Ashton KA, Talseth-Palmer BA, Rizos H, Zhang XD, Scott RJ, Hersey P (2011). P53 in human melanoma fails to regulate target genes associated with apoptosis and the cell cycle and may contribute to proliferation. BMC Cancer.

[42] Lu M, Breyssens H, Salter V, Zhong S, Hu Y, Baer C, Ratnayaka I, Sullivan A, Brown NR, Endicott J, Knapp S, Kessler BM, Middleton MR (2013). Restoring p53 function in human melanoma cells by inhibiting MDM2 and cyclin B1/CDK1-phosphorylated nuclear iASPP. Cancer Cell.

[43] Uramoto H, Wetterskog D, Hackzell A, Matsumoto Y, Funa K (2004). p73 competes with co-activators and recruits histone deacetylase to NF-Y in the repression of PDGF beta-receptor. J Cell Sci.

[44] Bolden JE, Peart MJ, Johnstone RW (2006). Anticancer activities of histone deacetylase inhibitors. Nat Rev Drug Discov.

[45] Costanzo A, Merlo P, Pediconi N, Fulco M, Sartorelli V, Cole PA, Fontemaggi G, Fanciulli M, Schiltz L, Blandino G, Balsano C, Levrero M (2002). DNA damage-dependent acetylation of p73 dictates the selective activation of apoptotic target genes. Mol Cell.

[46] Kato Y, Yoshimura K, Shin T, Verheul H, Hammers H, Sanni TB, Salumbides BC, Van Erp K, Schulick R, Pili R (2007). Synergistic in vivo antitumor effect of the histone deacetylase inhibitor MS-275 in combination with interleukin 2 in a murine model of renal cell carcinoma. Clin Cancer Res.

[47] Kulesz-Martin M, Lagowski J, Fei S, Pelz C, Sears R, Powell MB, Halaban R, Johnson J (2005). Melanocyte and keratinocyte carcinogenesis: p53 family protein activities and intersecting mRNA expression profiles. J Investig Dermatol Symp Proc.

[48] Vera J, Schmitz U, Lai X, Engelmann D, Khan FM, Wolkenhauer O, Pützer BM (2013). Kinetic modeling-based detection of genetic signatures that provide chemoresistance via the E2F1-p73/DNp73-miR-205 network. Cancer Res.

[49] Liu TC, Galanis E, Kirn D (2007). Clinical trial results with oncolytic virotherapy: a century of promise, a decade of progress. Nat Clin Pract Oncol.

[50] Cody JJ, Douglas JT (2009). Armed replicating adenoviruses for cancer virotherapy. Cancer Gene Ther.

[51] Höti N, Chowdhury W, Hsieh JT, Sachs MD, Lupold SE, Rodriguez R (2006). Valproic acid, a histone deacetylase inhibitor, is an antagonist for oncolytic adenoviral gene therapy. Mol Ther.

[52] Eberle J, Fecker LF, Forschner T, Ulrich C, Röwert-Huber J, Stockfleth E (2007). Apoptosis pathways as promising targets for skin cancer therapy. Br J Dermatol.

[53] Crighton D, O'Prey J, Bell HS, Ryan KM (2007). Crighton p73 regulates DRAM-independent autophagy that does not contribute to programmed cell death. Cell Death Differ.

[54] Gammoh N, Lam D, Puente C, Ganley I, Marks PA, Jiang X (2012). Role of autophagy in histone deacetylase inhibitor-induced apoptotic and nonapoptotic cell death. Proc Natl Acad Sci U S A.

[55] Wong SW, Tiong KH, Kong WY, Yue YC, Chua CH, Lim JY, Lee CY, Quah SI, Fow C, Chung C, So I, Tan BS, Choo HL, Rosli R, Cheong SK, Leong CO (2011). Rapamycin synergizes cisplatin sensitivity in basal-like breast cancer cells through up-regulation of p73. Breast Cancer Res Treat.

[56] Bisso A, Collavin L, Del Sal G (2011). p73 as a pharmaceutical target for cancer therapy. Curr Pharm Des.

[57] Maas AM, Bretz AC, Mack E, Stiewe T (2013). Targeting p73 in cancer. Cancer Lett.

[58] Zeng X, Overmeyer JH, Maltese WA (2006). Functional specificity of the mammalian Beclin-Vps34 PI 3-kinase complex in macroautophagy versus endocytosis and lysosomal enzyme trafficking. J Cell Sci.

[59] Tong Y, You L, Liu H, Li L, Meng H, Qian Q, Qian W (2013). Potent antitumor activity of oncolytic adenovirus expressing Beclin-1 via induction of autophagic cell death in leukemia. Oncotarget.

[60] Botta G, Passaro C, Libertini S, Abagnale A, Barbato S, Maione AS, Hallden G, Beguinot F, Formisano P, Portella G (2012). Inhibition of autophagy enhances the effects of E1A-defective oncolytic adenovirus dl922-947 against glioma cells in vitro and in vivo. Hum Gene Ther.

[61] Tormo D, Checińska A, Alonso-Curbelo D, Pérez-Guijarro E, Cañón E, Riveiro-Falkenbach E, Calvo TG, Larribere L, Megías D, Mulero F, Piris MA, Dash R, Barral PM (2009). Targeted activation of innate immunity for therapeutic induction of autophagy and apoptosis in melanoma cells. Cancer Cell.

[62] Rivera AA, Wang M, Suzuki K, Uil TG, Krasnykh V, Curiel DT, Nettelbeck DM (2004). Mode of transgene expression after fusion to early or late viral genes of a conditionally replicating adenovirus via an optimized internal ribosome entry site in vitro and in vivo. Virology.

[63] Quirin C, Rohmer S, Fernández-Ulibarri I, Behr M, Hesse A, Engelhardt S, Erbs P, Enk AH, Nettelbeck DM (2011). Selectivity and efficiency of late transgene expression by transcriptionally targeted oncolytic adenoviruses are dependent on the transgene insertion strategy. Hum Gene Ther.

[64] Luo J, Deng ZL, Luo X, Tang N, Song WX, Chen J, Sharff KA, Luu HH, Haydon RC, Kinzler KW, Vogelstein B, He TC (2007). A protocol for rapid generation of recombinant adenoviruses using the AdEasy system. Nat Protoc.

[65] Harms KL, Chen X (2007). Histone deacetylase 2 modulates p53 transcriptional activities through regulation of p53-DNA binding activity. Cancer Res.

[66] John K, Alla V, Meier C, Pützer BM (2011). GRAMD4 mimics p53 and mediates the apoptotic function of p73 at mitochondria. Cell Death Differ.

