# Supplementary material for: Eradication of metastatic melanoma through cooperative expression of RNA-based HDAC1 inhibitor and p73 by oncolytic adenovirus
Source: Oncotarget. 2014 Mar 21;5(15):5893–907. doi: 10.18632/oncotarget.1839 (PMC4171600; doi:10.18632/oncotarget.1839)
Supplement: Supplementary file 1 [file oncotarget-05-5893-s001.pdf]

SCHIPPER, ALLA ET AL: ERADICATION OF METASTATIC MELANOMA THROUGH COOPERATIVE EXPRESSION OF RNA-BASED HDAC1 INHIBITOR AND p73 BY ONCOLYTIC ADENOVIRUS.

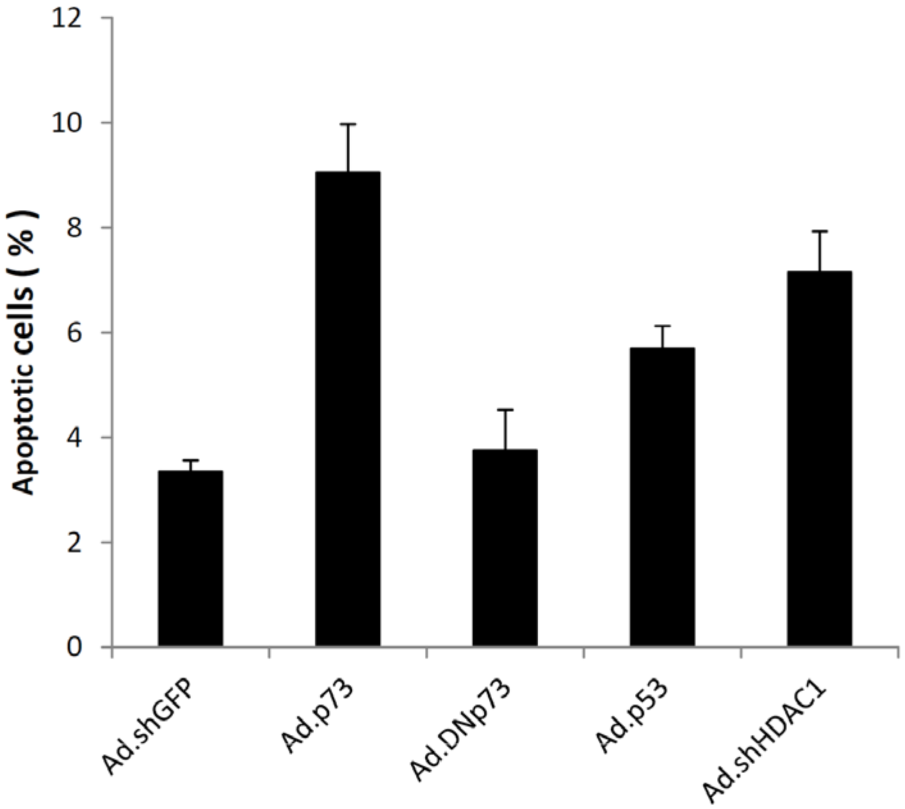

Supplementary Figure 1: Apoptosis induction by TAp73, DNp73, p53, and shHDAC1. The percentage of apoptotic SK-Mel-147 cells 48 hours after infection with Ad vectors expressing individual p53 family proteins or HDAC1 inhibitor was measured by FACS. Data are mean ± SD from two independent experiments.

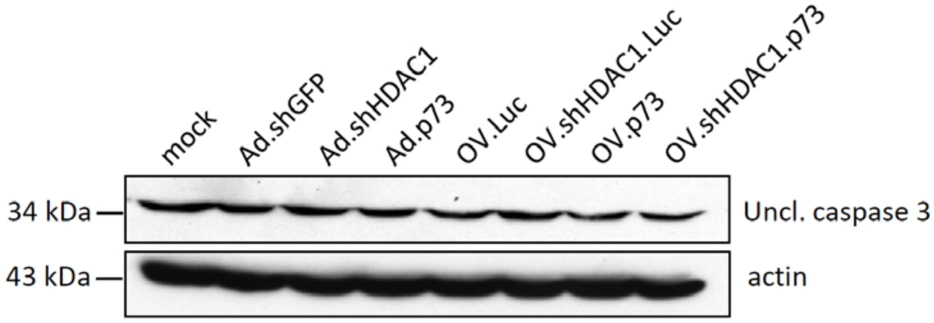

Supplementary Figure 2: Caspase 3 expression after virus infection. Detection of uncleaved caspase 3 protein in SK-Mel-147 cells infected with OVs and non-replicating Ad vectors at MOI 10 72 hours post-infection. Actin served as loading control.
